# Visual representations of single- and multi-sport participation in a youth swimming sample: Implications for definitions and discussions of early specialization

**DOI:** 10.1371/journal.pone.0292038

**Published:** 2023-09-27

**Authors:** Heather K. Larson, Bradley W. Young, Tara-Leigh F. McHugh, Wendy M. Rodgers

**Affiliations:** 1 Faculty of Kinesiology, Sport, and Recreation, University of Alberta, Edmonton, Alberta, Canada; 2 Faculty of Health Sciences, School of Human Kinetics, University of Ottawa, Ottawa, Ontario, Canada; 3 Faculty of Human and Health Sciences, University of Northern British Columbia, Prince George, British Columbia, Canada; University of Southern Denmark, DENMARK

## Abstract

Academic literature and sport policy documents have cited concerns about an increasing prevalence of early sport specialization, with associated burnout, dropout, and injury. However, evidence to support such statements is limited. Definitions of early specialization vary, but a common criterion is continued participation in a single sport, prior to adolescence. We explored the prevalence of single-sport participation and other patterns of sport involvement from ages 6–12 in a Canadian swimming sample using retrospective longitudinal methods. Parents of 236 competitive swimmers (ages 12–17) completed surveys on their children’s sport backgrounds, including the number of sports participated in annually from age 6–12. A cluster heat map elucidated single- and multi-sport patterns over time. Mixed analyses of variance tested for differences by gender and club type. Fourteen percent of our sample showed stable participation in either one sport or multiple sports per year over time, 25% decreased their annual number of sports, and 60% increased. This trend of increasing, rather than decreasing the number of sports in their annual activity roster when approaching age 12 was particularly pronounced for girls. Only 10 participants (4% of the sample) consistently engaged in a single sport each year from age 6–12. Summer (seasonal) swimmers consistently did more sports than year-round swimmers. Overall, our findings showed highly idiosyncratic longitudinal patterns of sport participation that did not easily conform to current sport activity guidelines. We also found similar idiosyncrasy in an ad-hoc analysis of participants who had dropped out of swimming a year later. If single-sport participation is considered a key criterion for defining early specialization, our findings suggest the prevailing narrative around early specialization may be overstated in relation to the number of single-sport athletes. Alternatively, other components of early specialization may be more prevalent and deserving of attention due to possible associations with harmful outcomes.

## Introduction

The organization Sport for Life includes the following statement on the Long-Term Development page of their website: “Kids and adults will get active, stay active, and even reach the greatest heights of sport achievement if they do the *right things* at the *right times* [emphasis added]” [1]. As is evident in literature pertaining to youth athletes and sport psychology, many researchers and policymakers believe doing “the right things” involves participation in the “right” number of sports. Broadly speaking, there are three possible trajectories of single or multi-sport participation over time: athletes can participate in a decreasing number of sports, an increasing number of sports, or the same number of sports as they get older. A great debate in youth sport is the age at which it is appropriate to specialize in one sport, to the exclusion of other sports and activities [2–6].

Researchers have cited an increasing prevalence of single-sport specialization prior to puberty with concerning health and psychosocial implications [7]. According to Smith, “We are currently in an era of early sport specialization” [8, p.220] and professional organizations such as the American Orthopaedic Society for Sports Medicine (AOSSM) [5] have released position statements largely condemning early specialization, citing potential associations with athlete burnout, dropout, and injury. Smith noted that early specialization—which she defined as “selecting a sport at an early age and pursuing it with singular focus” [8, p.221]—is not a new phenomenon; although the 1980s were when specialization in team sports became more common, early specialization in individual sports was happening in the 1950s and 1960s. This was especially true for girls, as their options for sport participation were more limited than those available to boys [8].

In 1993, Ericsson and colleagues [9] published research on the role of deliberate practice in talent development, which was extended to account for sport development trajectories [10]. Overly literal interpretations of their findings (e.g., Gladwell’s popularization of the “10,000 hour rule” [11]) advocated for earlier starts on single-sport specialization to maximize the amount of time needed to develop domain expertise. In response, there was a wave of critical sport-specific investigations of deliberate practice, with scholars expressing concerns around youth specializing in sport [12], and calling for multi-sport sampling as a remedy, citing its benefits [13].

Literature emerged supporting multi-sport sampling, some of which came to form the basis of the developmental model of sport participation (DMSP) [13,14]. These works built upon Côté [15], who conducted interviews with four elite athletes and their family members and noted distinct sampling, specialization, and investment phases of youth sport participation, which coincided with specific ages. The resulting DMSP suggested that sampling a variety of sports and activities from ages 6–12, then narrowing sport involvement to two sports around age 13 and investing heavily in one sport around age 15 or 16, would provide the best odds of attaining elite performance, without sacrificing health or enjoyment, and would facilitate long-term sport involvement. (Note that the ages associated with these DMSP stages were defined normatively, rather than being empirically based). In contrast, an early specialization pathway, while also potentially associated with elite performance, was proposed to carry greater risks of burnout, dropout and injury [16,17].

In the subsequent decade, an anti-specialization narrative grew, represented, for example, in Canada’s long-term development in sport and physical activity model (LTD 3.0) [18], which made specific recommendations about the number of sports in which children should participate at various ages: three sports per year from age eight or nine until puberty, and two sports per year for girls ages 11–15 and boys ages 12–16. In 2018, the Canadian Olympic Committee funded a nationwide multi-sport campaign, that included dire warnings about the risks of early specialization [19]. However, the research on early specialization has been criticized for lacking clear and consistent definitions and measurement of either specialization activity or outcomes [2,6,20]. Although attempts have been made to reach consensus on definitions of early specialization [5] and specialization in youth sport [21], such definitions have yet to be translated into operationalized measures [22]. This makes it difficult to ascertain the true prevalence, or indeed the effects of, early sport specialization.

In the United States, Buckley et al. [23] asked 3090 high school, collegiate, and professional athletes if they quit other sports to focus on one sport during childhood or adolescence. However, the term “adolescence,” an expansive life stage, was not defined. Of the high school athletes, 45.2% said yes, along with 67.7% of the collegiate athletes and 46% of the professional athletes. The average age at which participants reported quitting other sports to focus on one sport was 12.7 (*SD* = 2.4) for high school athletes, 14.8 (*SD* = 2.5) for collegiate athletes, and 14.7 (*SD* = 2.4) for professional athletes. This is earlier than recommended by the DMSP or LTD 3.0, but still not generally considered to be *early* specialization [5]. Another study in the United States [24] found that, of their sample of 1190 athletes between the ages of 7 and 18 years, 331 of them (26%) reported participating in only one sport and training in that sport for more than 8 months per year. These athletes were asked if they had ever quit other sports to focus on their main sport and 158 of them (13% of the total sample) responded affirmatively. The average age at which they did so was 12.0 (*SD* = 2.7) for team sport athletes and 11.2 (*SD* = 2.4) for individual sport athletes [24]. A limitation of both of these studies was that they did not account for athletes who only ever did one sport (rather than quitting other sports to focus on one), thereby missing some potential specializers. However, the latter study may have also oversampled early specializers due to their recruitment methods: 822 participants were injured athletes attending sports medicine clinics and only 368 were uninjured participants recruited from primary care clinics [24].

In a rare longitudinal study, Gallant et al. [25] collected data across five years, beginning with 756 Canadian children (55% girls; mean age of 10.7 years). According to the study definitions, at year 1, 14% of the children were considered sport nonparticipants, 67% were considered early samplers, and 19% were considered early specializers, defined as participating in one sport at least once a week for at least eight months out of the year. However, these “early specializers” could still participate in up to three other organized physical activities or sports for shorter periods of time throughout that same year, a pattern that is not representative of most definitions of early specialization. By year 5, 29% were considered sport nonparticipants, 44% were considered recreational participants (samplers), and 27% were considered performance participants (specializers). Their results suggested a general decline in the number of sports played with age, but again, the operationalization of sampling and specialization was generally inconsistent with early specialization literature.

Recent literature reviews have indicated that single-sport participation consistently appears as a key component of definitions of early specialization [20,26]. Therefore, failing to meet this unambiguous criterion would automatically preclude athletes from being categorized as early specializers by most definitions, without the need for any further data. Thus, the primary purpose of our exploratory study was to examine and visually represent the prevalence of single- and multi-sport participation from ages 6–12 years in a sample of over 200 Canadian athletes who were 12–17 years of age and involved in competitive swimming at the time of data collection. Swimming is one of the most popular sports among Canadian children [27] and is anecdotally considered an early specialization sport—a sport that one starts early and intensively, to the exclusion of other sports. Thus, this sample was well-suited for investigating the prevalence of single- and multi-sport participation at different ages and changes over time without biasing the sample by selecting based on a participation criterion or a dropout criterion. A more inclusive sample allows for observation of patterns of participation unlimited by pre-determined factors. Age 12 represents the end of the recommended sampling (multi-sport) years of elite athlete development in Côté et al.’s DMSP [13,14], meaning that most athletes should be engaging in multi-sport and not specializing up until this age.

A secondary purpose was to look for associations between patterns of sport participation by gender and club type. We pursued two research questions (RQs), both of which are addressed in our results:

**RQ 1:** What was the overall prevalence of single-sport participation and were there notable trends of within-athlete sport participation up until age 12?

**RQ 2.** Did these participation trends vary as a function of gender or club type?

## Methods

### Procedures

Upon receiving written approval from the University of Alberta Research Ethics Board 2 [ID: Pro00062507], we began recruiting participants from two different swimming contexts, which we refer to here as year-round and summer. Year-round swimming refers to the typical competitive swimming clubs that train almost year-round, from September until June or July, and tend to require high levels of investment and training volume. Summer clubs offer an alternative seasonal form of competitive swim programming that typically runs from May until August, providing more opportunities to participate in other sports and activities throughout the rest of the year. We began by first emailing the presidents and head coaches of summer swim clubs within the province of Alberta, which has a sizeable population of summer swimmers. If a club showed interest in participating in the study, the first author set up dates to visit and collect data in person. Swimmers completed their questionnaires before or after practice, and parents completed their questionnaires during practice. After the summer season concluded, we decided to use REDCap electronic data capture tools [28,29] to facilitate data collection and expand our reach beyond Alberta. We emailed the presidents and head coaches of year-round swim clubs across Canada and asked them to share the survey information and link with their members. In all, approximately 291 clubs were contacted directly, and 75 (29%) of them agreed to share the survey.

We ultimately recruited and surveyed a total of 265 competitive youth swimmers from Alberta, British Columbia, New Brunswick, Ontario, Quebec, Saskatchewan, and Yukon. Swimmers were between the ages of 12 and 17 at the start of data collection, with English proficiency to complete a questionnaire independently. Swimmers’ parents or guardians were given an information letter and the opportunity to ask questions about the study before signing a consent form. Swimmers gave written assent by signing the same form. Ongoing assent was implied by completion of the questionnaires.

#### Swimmer self-report

Swimmers indicated their gender, age, grade, ethnicity, and main sport—swimming, a different sport, or no single main sport. They also provided ratings on psychological variables (e.g., burnout, autonomy support) that were not included in this study but were analyzed in a larger project [see 6,30].

#### Retrospective questionnaire procedure for parents

Swimmers’ parents completed a complementary questionnaire on their child’s sport background, with a table to record the child’s yearly sport involvement from age 6 until the present. The questionnaire was based on a retrospective interview procedure developed by Côté and colleagues [31] and subsequently used in several studies of long-term athletic development [e.g., 17,32]. These studies found strong agreement between reports on training time by adolescent athletes and their parents, as well as with objective data from training logs. As parents often take responsibility for registering their children in sports and getting them to and from sport practices, there is precedent for using parental recall to obtain long-term retrospective sport participation data [33].

Parents indicated the total number of sports in which their child participated annually. For up to three sports each year, they were asked to name the sport and report the duration of the season in months, the average frequency and duration of practices or games per week, and whether the sport participation was competitive or recreational. Parents also provided demographic information, including their level of education and annual household income.

### Participants

The swimmers represented more than 50 competitive Canadian swim clubs. Sixty percent came from year-round clubs, and the remaining 40% came from summer clubs. Although it is possible to swim with a summer club and then switch to a year-round club or vice versa, the seasons overlap, so it is not possible to participate in both forms of programming every year. We did not have any participants who were involved in more than one swim club at the time of data collection. Of the year-round swimmers, about 95% indicated that swimming was their main sport, compared to 52% of summer swimmers. The remaining participants from both club types either said they had a main sport other than swimming or that they could not pick a single main sport.

The swimmers’ mean age at the start of data collection was 13.78 years (*SD* = 1.60), and their education level ranged from Grade 6 to 12, with 50% of the sample in Grades 7 and 8 (aged 12–14). The sample self-reported as 60% girls and 40% boys; none self-identified as trans or gender non-conforming. We asked about ethnicity, household income, and parental education to assess the representativeness of our sample as compared to the general population in Canada. Most participants (73%) self-identified as white, Caucasian, European, and/or Canadian. Fourteen percent of participants did not indicate ethnicity. The remainder reported Asian (4%), Indigenous (2%), or Hispanic/Latino (1%) descent, or belonging to more than one of these categories (6%). According to recent census data, 25% of people in Canada belong to racialized groups [34], and our sample likely fell short of reflecting that percentage. Most (67%) came from families with an annual household income of $100,000 CAD or more. For comparison, the median 2018 Canadian household income was $61,400 [35]. The parents were highly educated; 41% had bachelor’s degrees, and 28% had graduate or professional degrees.

### Data analyses

Our primary method of data analysis was a cluster heat map, described by Wilkinson and Friendly as, “an ingenious display that simultaneously reveals row and column hierarchical cluster structure in a data matrix” [36, p.1]. They noted two essential features: colour-shading, and matrix permutation to reveal underlying structures in data. From the parental questionnaire, we derived the total number of sports for each swimmer annually from age 6 to age 12. To create our colour-shaded matrix, we entered these data into an Excel spreadsheet, one participant per row, then colour-coded the cells with conditional formatting, based on the number of sports at each age. The darker the cell, the more sports they were engaged in that year. Of the original 265 respondents, there were 29 that had at least one cell of missing data. We used SPSS version 28 to run a Little’s MCAR test which revealed that these data were missing completely at random, χ^*2*^ = 49.33, *df* = 66, *p* = 0.94. Moreover, by cross-referencing neighbouring responses in the survey, we were certain that missing cells for the total number of sports were truly missing and not indicative of zero sports in a particular year. Therefore, we deleted these participants on a listwise basis to better visualize the patterns of sport participation. This left us with a complete set of descriptive data for the retrospective sport activity of 236 participants.

The main strength of the clustered heat map is that is “compacts large amounts of information into a small space to bring out coherent patterns in the data” [37, p.1772]. Therefore, our next step was to shrink and sort (permutate) the cells in exploratory ways to see the descriptive data and trends for all participants. We also examined the means and standard deviations for the number of sports at each age. Our exploratory analyses were tailored to our RQs:

*RQ 1*: We used a custom sort with seven levels to sort the full sample data in descending order by number of sports each year, beginning at age 6 and ending at age 12. Second, we did the same custom sort, but reversed the order of the levels to focus on where participants ended up at age 12. Third, we compared participants’ mean number of sports per year from ages 6–8 to their mean number of sports per year from ages 10–12.

*RQ 2*: We sorted the data based on gender and club type and conducted separate mixed analyses of variance (ANOVAs) in SPSS to look for within-group differences by age (6 years to 12 years), between-group differences, and interaction effects. The Greenhouse-Geisser correction was applied as needed to account for violations of sphericity. Effect sizes were based on partial *η*^*2*^ values interpreted as 0.01 small, 0.06 medium, and 0.14 large [38].

## Results

What was the prevalence of single-sport participation and were there notable trends of within-athlete participation up until age 12?

**Fig 1:**
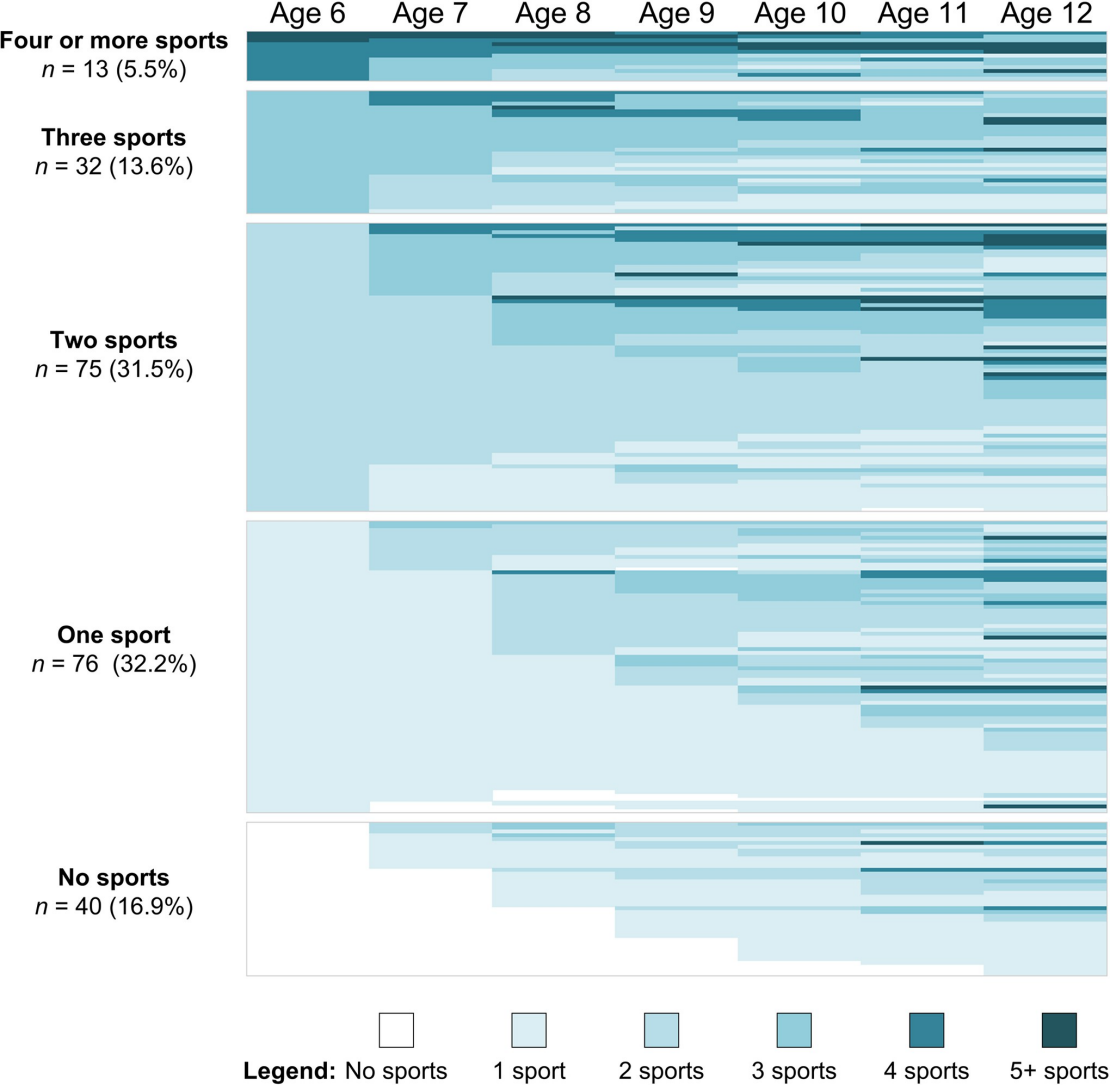
Longitudinal data for 236 participants sorted by number of sports at age 6. Participant data is presented in compressed colour-coded rows. Labels on the left-hand side represent profiles at age 6.

Fig 1 displays our first custom sort of the full sample of 236 participants. Although all participants were involved in competitive swimming at the time of data collection, their sport backgrounds were incredibly varied. This varied involvement is evident from the start. At age 6, approximately one-third (*n* = 76) were engaged in a single sport, another third were involved in two sports, close to 20% of the sample were doing three or more sports, and 17% of the sample of present-day swimmers were not involved in any sports. If one tracks those who were only doing one sport at age 6 over the years, only seven of the 76 athletes in this profile progressed to nonparticipation at some point. Again, all participants were engaged in sport at the time of data collection, so any participant who lapsed to nonparticipation along their trajectory did so temporarily and later returned to swimming. Forty-seven percent of the sample participated in swimming at age 6, 58% at age 7, 68% at age 8, 77% at age 9, 82% at age 10, 89% at age 11, and 95% at age 12.

**Fig 2:**
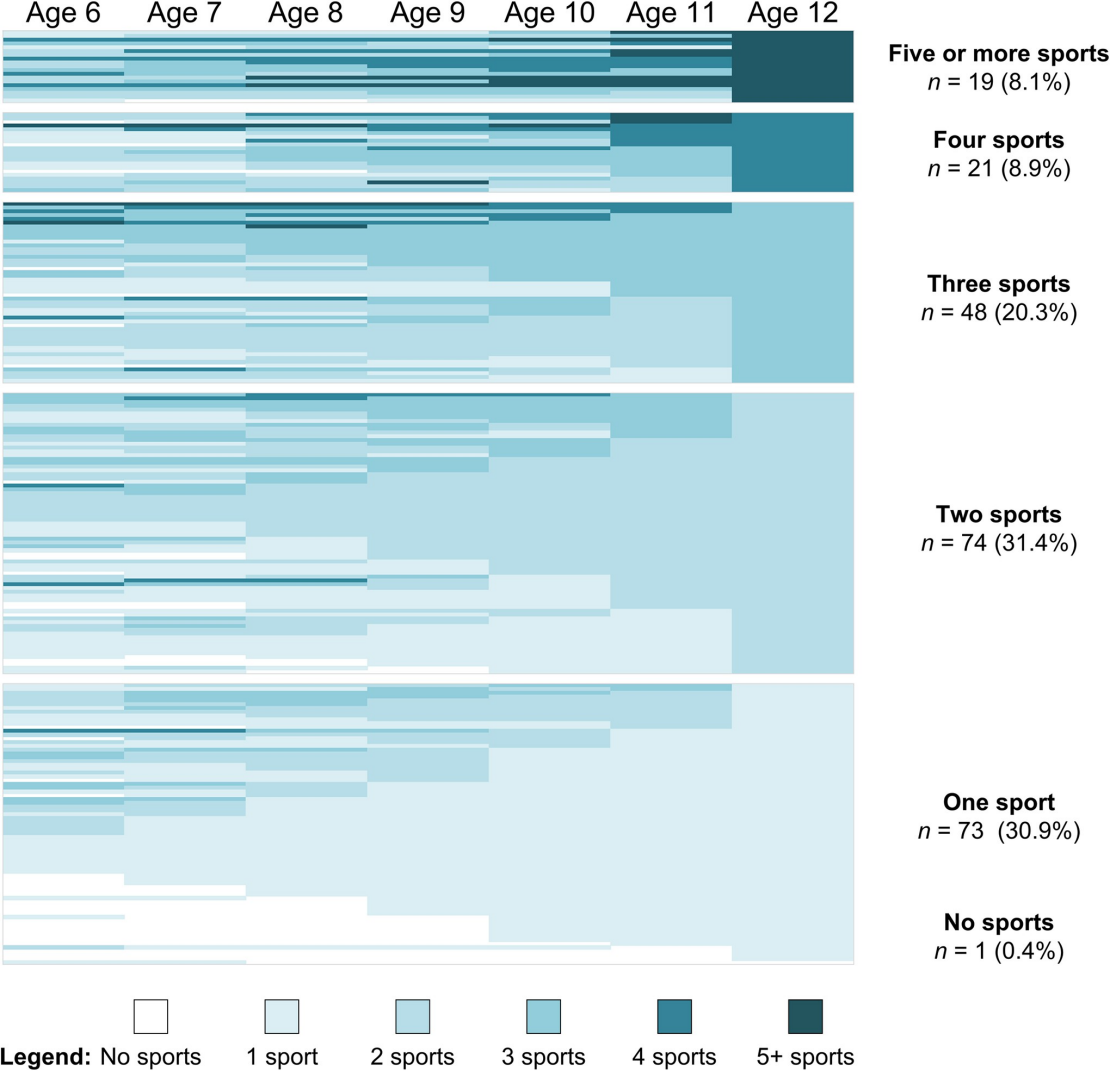
Longitudinal data for 236 participants sorted by number of sports at age 12. Participant data is presented in compressed colour-coded rows. Labels on the right-hand side represent profiles at age 12.

Fig 2 displays the reverse sorting of the full sample. At age 12, 31% of the sample reported being engaged in a single sport, with another 31% involved in two sports. Participation in three or more sports increased to 37% and there was just one nonparticipant who resumed sport involvement at the age of 13. From left to right, trends show relatively equal growth in the number of participants reporting “specialization” characterized by decreased involvement from multiple sports to one sport at age 12, compared to the number of participants who moved to take up two sports by age 12. It appears that almost equal numbers in this latter group, “two-sport participants” at age 12, entered this category after either increasing their participation from a single sport or decreasing from three or more sports.

**Fig 3:**
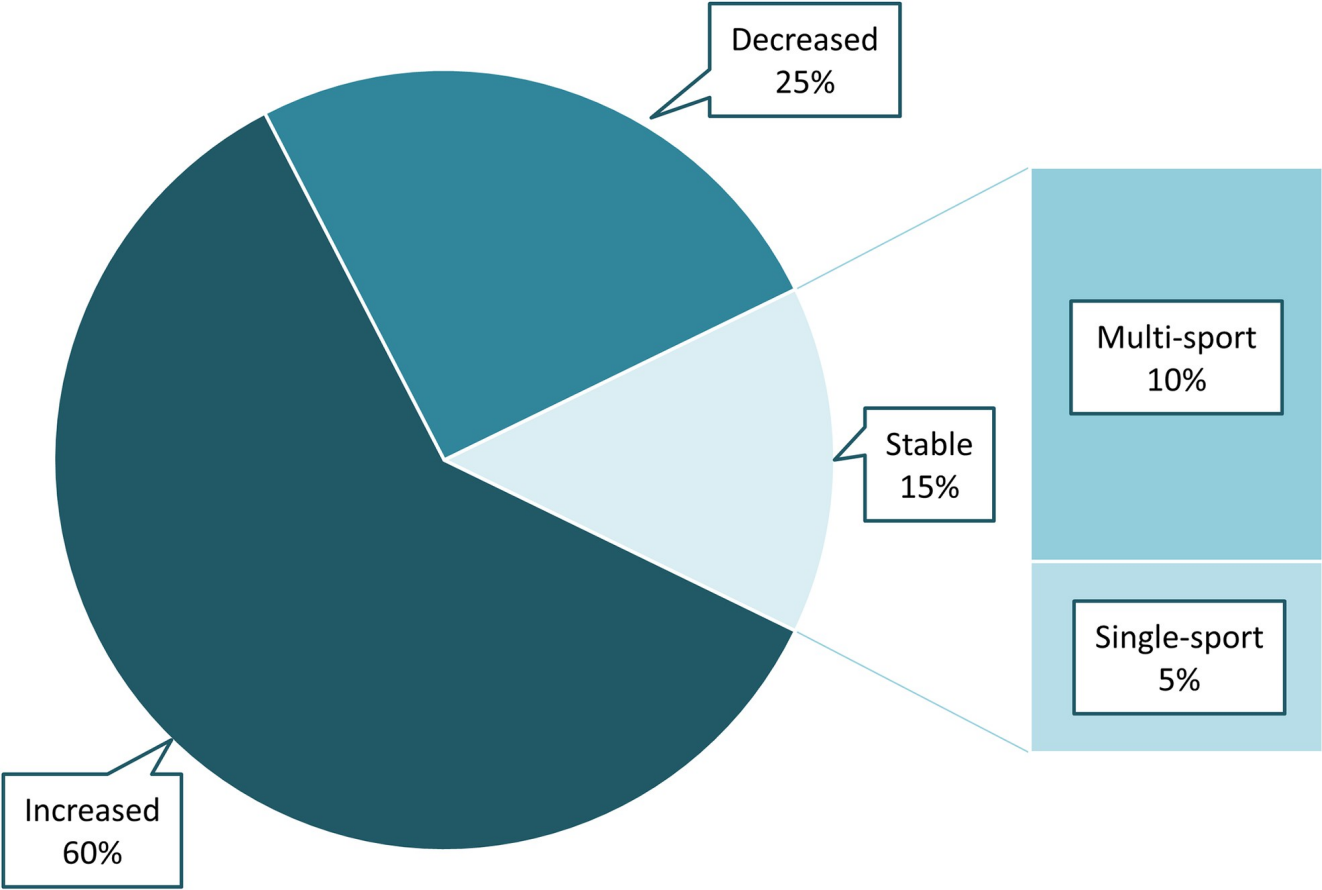
Categorization of general within-athlete participation trends from ages 6–12.

In our introduction, we noted three possible trajectories of single or multi-sport participation over time. Athletes can participate in fewer sports, more sports, or a stable number of sports as they get older. We categorized our participants by comparing their mean number of sports per year from ages 6–8 to their mean sports per year from ages 10–12. As shown in Fig 3, 25% of participants decreased their number of sports as they got older, 60% increased their number of sports, and 15% showed fairly stable participation in either one sport or multiple sports per year over time.

To focus on single-sport specialization, we examined the 33 participants (14% of the sample) who were reported as never having participated in more than one sport per year. Of those, 24 participated only in swimming. Most of the 33 participants had one or more years without any sport participation, but 10 participants (4% of the total sample) consistently engaged in a single sport each year from age 6 to age 12. We examined the data collected from their parents in more detail. Seven participated only in swimming from ages 6 to 12, and one participated only in soccer. The other two did only one sport per year, but that sport changed from year to year (e.g., from soccer at age 8 to swimming at age 9); they never engaged in two sports in the same year. By age 11, eight of the single-sport specializers were engaged in competition and the duration of their sport season was 9–11 months, but prior to this, some of them were still participating recreationally or for no more than 8 months of the year.

Did participation trends vary as a function of gender or club type?

Descriptive statistics by gender and club type are shown in Table 1.

**Table 1:** Mean (SD) Number of Sports From Ages 6–12.

		Age (years)						
	*n*	6	7	8	9	10	11	12
**Gender**								
Girls	143	1.59 (1.13)	1.72 (1.13)	1.80 (1.12)	1.87 (1.06)	1.95 (1.07)	2.13 (1.26)	2.52 (1.57)
Boys	93	1.60 (1.12)	1.66 (1.09)	1.95 (1.33)	2.13 (1.39)	2.00 (1.21)	2.02 (1.08)	2.18 (1.24)
**Club Type**								
Summer	90	1.91 (0.99)	2.04 (1.03)	2.17 (1.08)	2.30 (1.19)	2.20 (1.00)	2.36 (0.98)	2.89 (1.39)
Winter	146	1.40 (1.16)	1.48 (1.12)	1.66 (1.24)	1.77 (1.18)	1.83 (1.18)	1.92 (1.28)	2.08 (1.41)
**Total Sample**	236	1.60 (1.12)	1.69 (1.12)	1.86 (1.21)	1.97 (1.20)	1.97 (1.12)	2.08 (1.19)	2.39 (1.45)

### Gender

A mixed ANOVA for number of sport activities revealed that the main effect for gender was not significant, *F* (1, 234) = 0.002, *p* = 0.969, partial *η*^*2*^ = 0.00, observed power = 0.05. There was a medium-sized main effect for time, *F* (3.26, 763.45) = 23.80, *p* < 0.001, partial *η*^*2*^ = 0.092, observed power = 1.00 and a small interaction effect with gender, *F* (3.26, 763.45) = 3.49, *p* = 0.013, partial *η*^*2*^ = 0.02, observed power = 0.81. Parameter estimates showed that at age 9, the boys were engaged in an average of 2.13 sports, compared to the girls’ mean of 1.87, *t* = 1.64, *p* = 0.103, 95% CI [-0.053, 0.577]. However, by age 12, the girls were engaged in an average of 2.52 sports, having surpassed the boys’ mean of 2.18 sports, *t* = -1.74, *p* = 0.084, 95% CI [-0.714, 0.045].

#### Club type

A mixed ANOVA for number of sport activities showed a medium-sized main effect for club type, with summer (seasonal) swimmers consistently engaging in more sports than year-round swimmers, *F* (1, 234) = 17.97, *p* < 0.001, partial *η*^*2*^ = 0.07, observed power = 0.99. There was also a medium-sized main effect for time, *F* (3.25, 760.53) = 26.96, *p* < 0.001, partial *η*^*2*^ = 0.10, observed power = 1.00. The interaction effect was not significant, *F* (3.25, 760.53) = 1.81, *p* = 0.138, partial *η*^*2*^ = 0.01, observed power = 0.49.

#### Ad hoc dropout analysis

**Fig 4:**
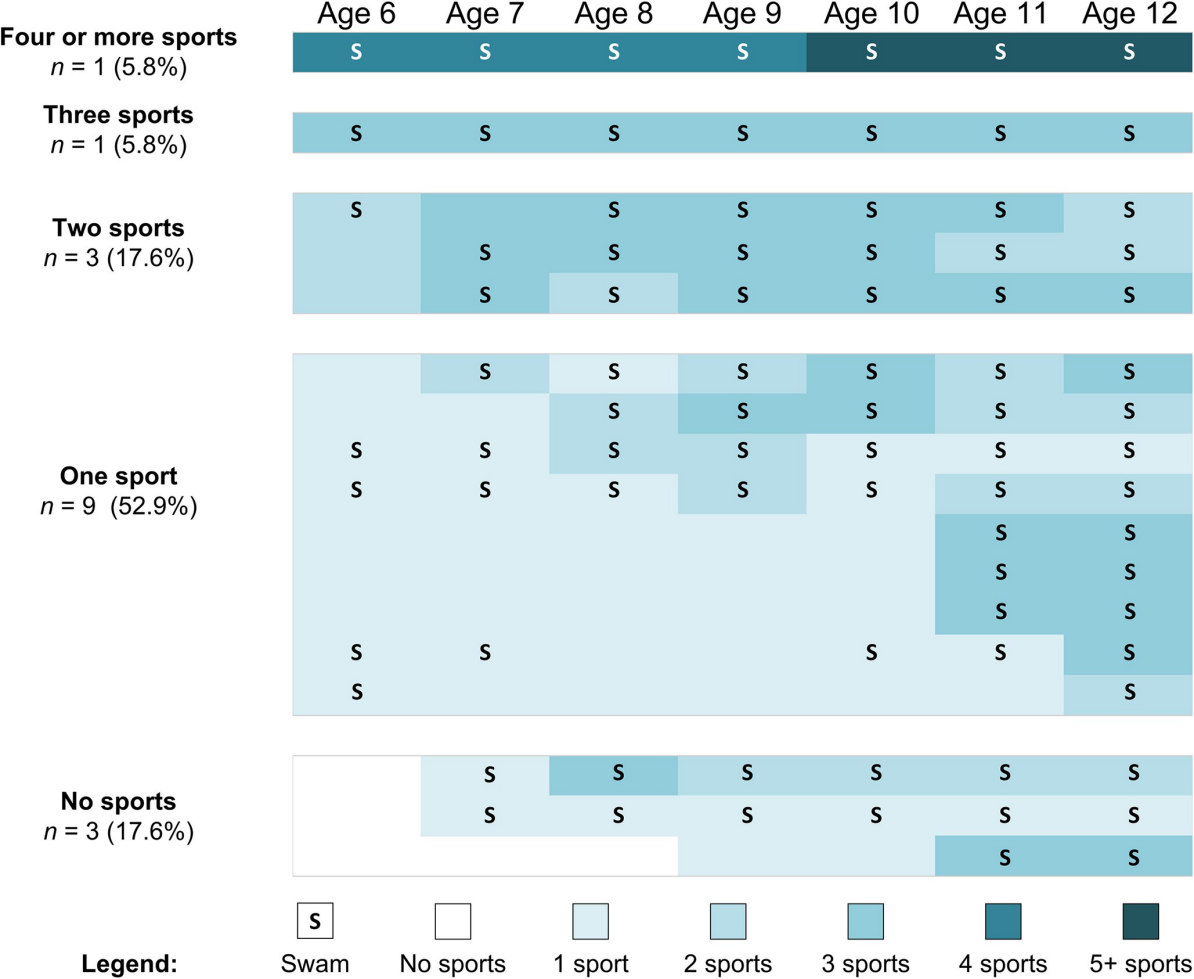
Longitudinal data for 17 participants in the swimming dropout subset sorted by number of sports at age 6. Labels on the left-hand side represent profiles at age 6. Swimming participation is denoted with the letter “S”.

We decided to bring in additional data from a follow-up survey that asked whether athletes were still swimming or not. This survey was sent to parents at the beginning of the subsequent swimming season—September, for year-round clubs, and May, for summer clubs. We received responses for all but 15 of our 236 participants. Parental reports indicated that 196 participants were still swimming, and 25 participants had stopped. We focused in on the 17 participants who were 12 or 13 at the time of initial data collection and then dropped out of swimming. This subset provided us with a sample for which we could observe whether single- or multiple-sport patterns from ages 6–12 were associated with sport-specific drop-out. We examined their patterns of participation and found that nine, or over 50% of them, were engaged in only a single sport at age 6 or 7 compared to around 30% of the total sample (see Fig 4). Furthermore, of the nine dropouts who began as single-sport participants at ages 6 or 7, they all took part in a sport other than swimming at some point before age 12. They all (with only one exception) progressed to involvement in two or three sports immediately before dropping out. Only two of the 17 dropouts were single-sport participants at age 12. None of the 17 dropouts showed an exclusive focus on one sport from ages 6 to 12.

## Discussion

There is limited empirical data precisely portraying the extent of early specialization, despite numerous claims of increasing early specialization and warnings about associated harms [5]. Single-sport participation is commonly used as a key criterion for measures and definitions of early specialization; therefore, our aim was to examine and visually represent the prevalence of single- and multi-sport participation from ages 6–12 years in a sample of over 200 Canadian youth swimmers. We identified 34 participants (14% of the sample) who never participated in more than one sport per year, and of these, a) just 24 (10% of the sample) participated only in swimming, and b) the other 10 participants, in some years, switched to a single sport that was not swimming. With respect to point a, one could take this to mean that the alarm over early specialization, particularly when predicated on single-sport participation, may be overblown—perhaps too much time, attention, and rhetoric is being given to an issue that is not all that prevalent in the sport system (at least in Canada and in our swimming sample). In terms of point b, it is unclear how such a pattern of single-sport switching (i.e., changing one’s single-sport involvement from year to year), as opposed to sampling multiple sports simultaneously, should be interpreted, as it is not acknowledged by popular youth sport models or policy documents.

Youth sport models and literature, both academic and policy-based, continue to prescribe certain sport pathways that will lead to certain outcomes—whether elite performance, dropout, or continued recreational participation. Our findings around activity patterns portray idiosyncratic pathways that lack such easy definition. Our participants showed remarkable diversity in their sport backgrounds. Less than 4% demonstrated patterns of sport participation that corresponded with the number of sports recommended at certain ages by Canada’s LTD document: three sports per year from age eight or nine until puberty, and two sports per year for girls ages 11–15 and boys ages 12–16 [18]. The majority increased, rather than decreased their annual roster of sports as they got older, contrary to the general trends described in the DMSP [12,13].

Although swimming is considered an early specialization sport, single-sport participation did not appear as a problematic activity trend within our sample. In fact, only seven of our 236 participants showed a stable pattern of swimming-only participation all the way from age 6 to age 12. This finding is consistent with other studies that have found low rates of early single-sport participation among athletes in North America, Europe, and other parts of the world [39,40]. Although the single-sport criterion for early sport specialization is clearly not concerning in our sample, it is possible that more of our swimmers were experiencing early specialization based on other criteria. Indeed, Mosher and colleagues cautioned “the harmful mechanisms behind early specialization are undoubtedly more complex than just single-sport participation” [20, p.19]. There could have been other more important facets of early specialization, specifically related to intensity and its implications on contextual and relational factors, that we left unexamined.

Simplistic measures of early specialization that do not adequately capture intensity of involvement and the psychosocial context in which training takes place leave us with inadequate information for making specific recommendations regarding best practices for single- and multi-sport participation at population levels. A qualitative study of 11 sport parents with scholastic expertise in sport psychology and coaching science lends support to this assertion [41]. The study revealed that these parents made decisions around their children’s competitive sport involvement that were not focused on activity indices (the right number of things at the right times), but were instead based on surveilling, evaluating, and modifying the sport context to best support their children and their families. In that study, although the sport parents did not advocate for a single sport focus, multiple-sport participation in organized sport was seen in variable and family-specific terms, and not always in a positive light [41].

The primary analyses of our study were descriptive. They were restricted in terms of associating some of these descriptive trends (however diverse they were) with implications for sustained participation or dropout. The value of the analyses for the subset of 17 dropouts was to look at whether there was overrepresentation of single-sport participation or multi-sport participation on whether people continued a year on in swimming. Even here, no concrete interpretations can be made. We did see overrepresentation of single-sport participation early on in the dropouts, but of the nine dropouts who started with single-sport swimming, the vast majority dispersed into multiple sports before dropping out of swimming. Fraser-Thomas et al. [32] also compared the sport backgrounds of engaged adolescent swimmers to participants who dropped out of swimming between the ages of 14 and 17. Although they found that engaged swimmers had a significantly higher number of extra-curricular activities (e.g., drama or music lessons) per year compared to dropouts, there were no significant differences in their number of sports per year. The dropouts averaged 3.2 sports per year from age 6–10, and 3.8 sports per year from age 11–12. Although this number decreased after age 12, they were still averaging at least 2.8 sports when they withdrew from swimming. A similar study of hockey players by Wall and Côté [42] indicated that “both the active and dropout players enjoyed a diverse and playful introduction to sport” (p.1). With these studies in mind, we interpret Côté and Vierimaa’s [43] reference to “a strong association between early specialization and increased sport attrition,” (in which both of the aforementioned studies were cited), as referring to aspects of early specialization other than single- vs. multi-sport involvement (p.S65).

A secondary purpose of our study was to examine trends by gender. The Rally Report [44] cites the percentage of Canadian girls participating in weekly sport as only 47% for 13-15-year-olds and 38% for 16-18-year-olds. The participation patterns of the adolescent girls in our sample, who were a part of this minority, may offer insights for improving the retention of girls in sport. Of note is the increase in multi-sport participation from age 11 to 12, which in Canada may be athlete-driven and related to opportunities provided by school sports rather than club sports [45]. This transition period may represent a critical juncture, where increasing (rather than decreasing) participation in multiple sports may lead to greater protection against dropout down the road, for the same reasons sampling is recommended at younger ages: to gain competence in multiple movement domains, more opportunities for positive youth development, possible protection against injury (particularly overuse injuries), and a chance for a developing athlete to find “their sport.” Although we noted a medium-sized main effect of time for our whole sample, this trend was more pronounced for the girls, as they did fewer sports than boys at age 9 but seemed to reach and even surpass boys at age 12. Further evidence for the potential benefits of secondary sampling for adolescent girls comes from a scoping review of elite female athletes’ developmental pathways. Peters and colleagues [46] found that elite female athletes who accumulated high volumes of practice in their main sports early on continued to engage in multiple sports through adolescence, with some showing age-related increases in diversified sport involvement. It may be that secondary sampling in early adolescence protects against dropout from sport later on, allowing for the development of elite performance.

Our exploration of participation trends encompassed adolescent swimmers from two contexts: summer and year-round. Summer swimmers consistently engaged in more sports than the year-round swimmers, who predominantly reported that swimming was their main sport. Upon seeing these data, Eys [47] noted that,

…if individuals want to keep diversification going throughout childhood then they [may] need to start with a wide variety of activities early on. However, if they (a) start sport participation at a later age or (b) do not get exposure to a variety of activities early on, then perhaps they are forced to catch up with a particular activity [by specializing].

We think this is a plausible interpretation, and we also wonder if participants’ initial swim club experience might also be highly influential. For example, if a child’s first competitive swimming experience is with a summer club, and they like it, they may be more likely to continue on in summer club and find other sports to engage in for the rest of the year. However, if their first exposure is to a year-round swim club, and it is positive, they will likely continue on with that club and prioritize swimming as their main sport, resulting in increasingly limited time for other sports.

### Limitations and future directions

We believe our sample of Canadian swimmers has merit because of the country’s prevailing policy narrative, which is anti-specialization and highly supportive of multi-sport, and because Canada is a high-performing country in swimming. Although one could argue that this focus limits the generalizability of our findings, it is worth noting that the Canadian LTD document [18] has been introduced to sport organizations and sport managers in over 65 countries [48]. Thus, we believe readers may consider our findings in relation to the development trajectories in their own countries. With respect to our methods, we only examined the sport backgrounds of participants who were currently engaged in sport (swimming) at the time of our initial data collection. This sample included some sport-specific dropouts (participants who did not continue swimming), but we did not specifically ask about new or continued involvement in other sports. Therefore, we cannot speak to the trajectories and backgrounds of nonparticipants or general sport dropouts. Although we found many patterns of single and multi-sport engagement that all resulted in continued sport participation in adolescence, it may be that nonparticipation in sport is predicted by a few distinct childhood patterns, and plausibly by specialization at earlier points.

Further research using prospective longitudinal designs is needed, including modeling that distinguishes sport dropouts from those who persist or transfer to a different sport. This would be valuable in determining whether concerns are warranted about single-sport aspects of specialization having a negative impact on long-term persistence in sport. The prospective nature of such a design is important because the selection of only dropouts in a retrospective design, for example, does not allow for assessment of the prevalence of dropout versus sport switching. Furthermore, retrospective designs may limit the interpretation of diverse developmental variables because they typically involve interviewing and/or surveying participants defined by some important criterion (e.g., athletes who are presently on national teams and are asked to divulge activities for how they got there), which may inadvertently result in samples that are more homogenous in their experiences than developmental cohorts that could be tracked over time (see Baker et al.’s [49] comments on survivorship bias). Such studies should include reliable assessment of involvement and the extent to which sport activities are formal and organized (which is rarely considered in retrospective longitudinal accounts), measures of training volume in various sports (which were not considered in the present study), and psychosocial contextual factors as potential moderators. Another interesting topic for future research is the prevalence of and outcomes associated with single-sport switching (e.g., only soccer at age 6, followed by only gymnastics at age 7), as theoretical frameworks, policy papers, and ad-hoc definitions of “early specialization” in the literature typically do not consider this pattern. Neither do they consider synergistic combinations of specific sports (what could be termed multi-sport combinations) and their potential benefits. It is also worth exploring the drivers of secondary sampling for girls, particularly in relation to school sports, as this could provide an avenue for future interventions.

## Conclusion

Overall, we noted a low prevalence of single-sport participation in our adolescent swimming sample. Our data describe diverse trajectories, and remarkably little evidence of concentrating on or limiting involvement to a single sport before age 13. Our findings suggest that the energy and text we allocate to blanket admonition of early specialization may be misdirected and perhaps more attention should be paid to scanning for and identifying those exceptional cases of athletes who are experiencing unhealthy physical and psychosocial sport contexts and outcomes. The criteria for early specialization may need to put less weight on single-sport participation and focus more on other potentially harmful aspects. Athlete-driven multi-sport participation may be helpful in promoting long-term participation, particularly for girls, by providing increased opportunities to experience positive relationships and feel competent. Although the majority of our sample increased their average sports per year from ages 6 to 12, we also noted that participants involved in summer swimming consistently engaged in more sports per year over time compared to those who were involved in year-round swimming. An early commitment to a particular sport organization or main sport may have lasting impacts on the size of a child’s sport roster across early adolescence, unrelated to a conscientious choice to limit or specialize. Further research can examine these decision-making processes.

## Supporting information

S1 Dataset Data for 236 participants. (XLSX)
